# Dorsal root ganglion stimulation for treatment of complex regional pain syndrome of the foot

**DOI:** 10.3171/2020.7.FOCVID2034

**Published:** 2020-10-01

**Authors:** Kevin Hines, Fadi Al Saiegh, Aria Mahtabfar, Kavantissa M. Keppetipola, Caio M. Matias, Chengyuan Wu, Ashwini Sharan

**Affiliations:** Department of Neurosurgery, Thomas Jefferson University and Jefferson Hospital for Neuroscience, Philadelphia, Pennsylvania

**Keywords:** dorsal root ganglion, neuromodulation, neurostimulation, complex regional pain syndrome, foot pain

## Abstract

This is a case of a 54-year-old man presenting with complex regional pain syndrome (CRPS) type 1 of the right lower extremity, which was most debilitating in the plantar aspect of the right foot. The patient had prior treatment with thoracic spinal cord stimulation; however, the foot pain remained intractable. Given that his pain was predominantly in his foot and remained debilitating despite thoracic spinal cord stimulation, it was recommended that the patient undergo a trial of dorsal root ganglion (DRG) stimulation. The surgical technique for placement of dorsal root ganglion stimulators is demonstrated in this operative video.

The video can be found here: https://youtu.be/_1xMxFZa6tU

## Transcript

**0:24 Case Presentation.** This is a case of a 54-year-old male presenting with right lower-extremity pain secondary to CRPS. He previously had implantation of a percutaneous thoracic spinal cord simulator with moderate relief. However, he still continued having debilitating plantar allodynia not adequately addressed by the spinal cord stimulator. Given its relative efficacy compared to spinal cord stimulation for targeting foot pain^1–3^ in patients with CRPS, percutaneous dorsal root ganglion stimulation trial was recommended for this patient. The mechanism of DRG generation of chronic pain has been postulated to involve ectopic DRG firing, altered gene expression of ion channels, and modification of growth factors contributing to pathologic sensitization to pain.^4–7^

**1:04 Demonstration of Positioning.** For placement of DRG of the low lumbar or sacral levels, the patient is positioned prone on a Jackson table with a Wilson frame. This allows fluoroscopic visualization of the lowest levels while maximally opening the interlaminar spaces for optimal access to the epidural space for electrode placement. Before prepping and draping, the pedicles of the target levels are marked on true AP views to aid in planning entry points. Based on institutional experience, lead placement is confirmed by anatomical landmarks on fluoroscopy under general anesthesia for patient comfort.

**1:30 Entry Point for L5 Electrode.** Before prepping and draping, the pedicles of the target levels are marked on true AP views to aid in planning entry points. Entry points for lumbar levels start inferior and contralateral to the target neural foramen. This facilitates catheterization of the foramen after passing through the interlaminar space. The entry point is chosen so that as the needle approaches the interlaminar space on an AP view, it approaches midline.

**1:44 Entering Epidural Space With Guide Needle.** After confirming lateral to medial trajectory on an AP, the fluoroscopy is flipped to a lateral view to visualize the approach to the spinal canal. As the needle is about to enter interlaminar space, a loss-of-resistance syringe is used to confirm entry into the epidural space.

**2:11 Catheterizing Entry Into the Spinal Canal.** Upon confirmation of entry into the epidural space, the loss-of-resistance syringe is removed and the guide catheter, containing a guidewire, is advanced through the guide needle. When the needle and catheter is seen in the spinal canal, the fluoroscopy view may be changed to an AP view.

**2:25 Catheterization of the Right L5 Foramen.** The needle bevel and guide catheter curl (denoted by the direction of clear plastic tubing) are pointed to the patient’s right, toward the foramen. The catheter is advanced under the L5 pedicle into the dorsal neural foramen.

**2:41 L5 Electrode Placement.** After entering the dorsal aspect of the neural foramen, the guidewire is used to advance the electrode. The electrodes are advanced over the DRG on AP fluoroscopy.

**2:45 Development of Superior Retention Loop.** To prevent lead migration, superior and inferior retention loops are formed. This is accomplished by retracting the guide catheter by several millimeters, aiming the guide catheter and needle bevel superiorly toward the patient’s head, and then advancing the electrode to form the loop. The guidewire may also be partially retracted into the needle so the softer electrode may coil into a superior loop.

**3:17 Development of Inferior Retention Loop.** With the guidewire still partially retracted, the needle bevel and guide catheter are directed inferiorly, toward the patient’s feet. The electrode is then advanced until an inferior retention loop is formed. Dorsal position of the electrode within the foramen is confirmed on lateral fluoroscopy. At this time, the guidewire, catheter, and needle are removed while providing counterforce on the electrode to prevent migration.

**3:53 Entry Point for Right S1 Electrode.** Since S1 foramina access does not require an interlaminar trajectory and the foramina are more coronally oriented than the sagittally oriented lumbar counterparts, an ipsilateral, inferior entry point is utilized to approach the foramen on AP fluoroscopy.

**4:10 S1 Electrode Placement.** In patients with high sacral slope, a less inferior entry point to the S1 pedicle may be required to effectively pass through the foramen with the guide catheter. Here, the patient’s anatomy made entering the foramen difficult, so a needle was left in S2’s foramen as a placeholder. Once the S1 foramen has been accessed, the guide catheter with guidewire and electrode is advanced under lateral fluoroscopy. This is initially done with the needle bevel and guide catheter directed inferiorly.

**4:54 S1 Electrode Placement Confirmation.** After using the guide catheter to advance the electrode, confirmation of placement may be obtained on AP fluoroscopy. The electrode may then be seen entering the S1 foramen and passing under the S1 pedicle. On lateral fluoroscopy, the surgeon should aim to have one electrode contact anterior to the sacral border with the majority of contacts posterior and along the sacral border. This will capture the typical intracanalar and intraforaminal locations of the S1 DRG.^8^

**5:02 Development of Retention Loops.** After the electrode overlies the right S1 DRG, the needle and guide catheter may be directed superiorly. At this point, the electrode is advanced until a superior retention loop is formed. Similarly to the lumbar electrode, the needle bevel and guide catheter may be directed inferiorly while advancing the electrode to form the inferior retention loop. After development of retention loops, guide catheter, wire, and needle are removed. Fluoroscopy may be used to confirm satisfactory location of lead placements after each piece is removed. Given the patient’s anatomy and technical challenges associated with DRG placement, surgeon preference was to perform a buried trial with lead extensions. This allows for conversion to a permanent implant while stimulating the exact same location as the trial should the patient obtain adequate pain relief during the trial period.

**5:50 Lead Tunneling.** A pocket for extension connectors is created adjacent to the future battery site, and a Tuohy needle is used to tunnel the L5 and S1 DRG electrodes to this site.

**6:05 Extension Placement and Tunneling.** Leads are inserted into extension connectors, and the screw mechanism is used to lock them into place. Retention loops are sutured into place within the pocket to provide strain relief.

**6:28 Impedance Check.** The extensions are then hooked up to the interrogation system to ensure integrity of the system after tunneling and connecting to lead extensions. This is done in real time in the operating room to confirm placement prior to ending the procedure.

**6:33 Closure and Dressing.** All incisions are closed in two layers, including dermal and simple skin layers. Incisions are dressed with Telfa gauze pads and covered by Tegaderm.

## Acknowledgments

We would like to thank the operating room staff involved in the case for accommodating the changes necessary to ensure high-quality recording of the procedure.

## Author Contributions

Primary surgeon: Sharan. Assistant surgeon: Hines. Editing and drafting the video and abstract: Hines, Al Saiegh, Mahtabfar, Keppetipola, Wu. Critically revising the work: Hines, Al Saiegh, Mahtabfar, Keppetipola, Matias. Reviewed submitted version of the work: Hines, Al Saiegh, Mahtabfar, Keppetipola, Matias, Wu. Approved the final version of the work on behalf of all authors: Hines. Supervision: Matias, Wu, Sharan.
